# Supplementary material for: Are carbon emissions trading and green financial instruments synergistic? -Comprehensive quantitative research based on content analysis
Source: PLoS One. 2024 Mar 7;19(3):e0298601. doi: 10.1371/journal.pone.0298601 (PMC10919601; doi:10.1371/journal.pone.0298601)
Supplement: S1 Data — (ZIP) [file pone.0298601.s001.zip › Data/地级市Z_F工作报告.zip!/─┌├╔╣┼/╬┌└╝▓∞▓╝╩╨/└┤╫╘╣1⁄2╓┌║┼í╛┬φ┐╦╩2╛▌═°í┐_2017.docx]

各位代表：

现在，我代表市人民政府向大会报告工作，请予审议，并请市政协委员和列席会议的同志们提出意见。

一、2016年工作回顾

过去一年，是我市发展史上极不平凡、极富挑战的一年。面对各种困难和风险考验，在自治区党委、政府和市委的坚强领导下，全市各族人民以坚定的信心和非凡 的勇气，攻坚克难，开拓进取，有力推动了中央、自治区各项决策部署在乌兰察布落地生根、开花结果，实现了“十三五”良好开局。预计完成地区生产总值955 亿元,增长6%；一般公共预算收入56.7亿元，按可比口径增长8.3%；固定资产投资672亿元，增长2%；社会消费品零售总额316亿元，增长9%； 城乡居民人均可支配收入分别达到26565元和9191元，增长8％和9％。

——坚持项目带动，产业特色更加鲜明。秋季重大项目集中开工建设动员大会，打响了重大项目建设攻坚战，全年实施亿元以上项目280个，10亿元以上项目 34个，39个项目列入国家和自治区PPP项目库，社会融资规模达到1000亿元，新雅宝路、大北农等一批大项目纷纷落地，为经济稳健增长提供了有力支 撑。现代农牧业稳步推进。马铃薯面积稳定在400万亩，产量增长36%。冷凉蔬菜新增7万亩，达到85万亩。燕麦面积扩大到100万亩，较上年翻了一番。 牧业年度牲畜总头数910万头（只）。农畜产品加工转化率达到56%。甘蓝等3个蔬菜新品种获得国家认证，实现自主知识产权“零”的突破。“后旗红”马铃 薯荣获第十四届中国国际农交会金奖。乌兰察布莜麦通过国家地理标志认证。工业经济实现提质增效。千万千瓦级能源基地全面建成，电力总装机达到1340万千 瓦。氟化工、氯碱化工、石墨新材料产能分别达到25万吨、89万吨和29万吨，中国首条年产30吨薄层石墨烯生产线建成投产。铁合金产量占全国的11%。 大数据产业迅猛发展，华为云数据中心启动运行，举办大数据产业北京推介会，成功加入中关村“一司一金一中心”合作联盟体系。“三个交易中心”实现营业收入 96.5亿元。开通乌兰察布——北京旅游专列，新增4A级景区4个，接待游客1420多万人次，实现旅游收入122亿元，增长22%，创历史新高。首届国 际露营大会、中国创业创新博览会、全国图书交易博览会和乌兰察布首届冰雪节等一批品牌展会成功举办，会展经济蓬勃兴起。养老、体育等新兴服务业发展势头强 劲。

——致力先行先试，发展活力加速释放。习近平总书记要求内蒙古先行先试的三项改革深入推进，农企紧密型利益联结比例达到35％，四子王旗国家主体功能区 建设取得进展，化德县进入国家重点生态功能区。中旗土地确权试点工作基本完成，四子王旗草原确权和中旗、后旗基本草原划定工作通过自治区验收。土地流转面 积达到40%以上，居全区前列。全面落实“三去一降一补”任务，退出60万吨粗钢产能。商品房待售面积下降31%。取消和免征涉企收费20项，累计为企业 降低用电成本22.6亿元。最大用电负荷426万千瓦，创历史新高。“放管服”改革成效明显。市本级取消和下放行政权力140项，完成496项依申请项目 流程再造，审批环节减少27%，市县两级责任清单向社会公开。开通网上办事大厅，市县乡村四级政务服务网络实现互联互通。建立重大项目审批“绿色通道”， 企业和群众办事时间压缩一半以上。设立全区首家权力监督管理办公室，“双随机一公开”监管稳步推进。商事制度改革不断深化，“五证合一、一照一码”全面推 行，个体工商户“两证整合”正式启动，市场主体增长8%。不动产登记全面实现“发新停旧”。财税金融改革扎实推进，“营改增”试点全面推开。

对外开放水平不断提升。长城金三角合作日益紧密，20项合作协议有效落实。京蒙对口帮扶成效显著，融入京津冀步伐加快，签署项目139个、总金额达 793亿元。13家医院、48所中小学与北京建立了合作关系。天津自贸区进口商品及平行汽车直营中心建成运营。向北开放迈出新步伐，中欧班列枢纽节点城市 成功获批，乌兰察布——阿拉木图班列开通运行。集宁海关实现通关便利化，进出口总额8000万美元，增长8%。

——推进城乡统筹，人居环境明显改善。“五城联创”成果丰硕，国家园林城市成功创建，国家食品安全城市创建工作成为全国试点，国家卫生城市顺利通过暗访 考评，全国文明城市通过第二年度测评，国家环保模范城市创建取得阶段性成果。丰镇等6个旗县市创建为自治区园林县城，中旗等3个旗县创建为自治区卫生县 城。城市发展实现由外延式扩张向内涵式提升的重大转变，城镇化率达到48.3%。中心城区“多规合一”试点工作启动。实施水电路气讯等城建项目565项， 市政公共设施建设水平进入全区前列。棚户区改造1.66万套，超额完成年度任务。城镇绿化工作提档升级，新增绿化面积430万平方米。中心城区实现 “300米见绿、500米见园”的园林景观效果，人均公园绿地面积28.6平方米。农村牧区面貌焕然一新，完成危房改造4.1万户,超目标任务1.1万 户。完成1321个行政村、2307个30户以上自然村的公共服务建设任务。

14 个镇入选全国重点镇，4个村入选中国传统村落名录。基础设施全面加强，公路建设完成投资100亿元，建设规模1300公里，居全区首位。基本实现嘎查村沥 青水泥路全覆盖。京呼高铁乌兰察布段全面铺轨。乌兰察布机场通航城市达到14个。3座中型水库主体完工。“宽带乡村”建设列入全国电信普遍服务试点地区。 实施国家和地方生态建设工程124万亩，森林覆盖率达到23.1%。启动岱海、黄旗海水生态保护治理工程，生态自然修复能力明显提高。

——不忘奋斗初心，民生工作全面加强。全年财政用于民生的支出占一般公共预算支出的84%。全面打响脱贫攻坚战，实施“五个一批、六个精准”，13.7 万贫困人口全部建档立卡。发放金融扶贫贷款16.4亿元。撤并小村877个，开工建设易地搬迁安置点645个。贫困人口全部纳入大病医疗救助范围。全市3 万贫困人口实现稳定脱贫。统筹推进就业创业，建成就业园14个、创业孵化基地12个。3家众创空间被认定为国家级众创空间。农村牧区劳动力转移就业36万 人，城镇登记失业率控制在3.8%以内。社会保险扩面任务超额完成。企业退休人员养老保险待遇平均增幅提高6.5%。社区居家养老服务工作不断拓展。在呼 包两地实现异地参保与就医即时结算。城乡低保实现动态管理下的应保尽保。

社会事业全面进步。提前一年完成第二期学前教育三年行动计划，5个旗县通过国家义务教育发展验收。3所学校成为“自治区民办示范学校”。乡村教师队伍素 质提升工程被教育部确定为全国优秀工作案例。民族教育、特殊教育、职业教育和高等教育加快发展。健康乌兰察布建设稳步推进。被确定为第四批城市公立医院改 革国家试点城市，市县两级公立医疗机构全部取消药品加成，并调整了医疗服务价格。市120急救指挥中心正式启动，市中心医院晋升三甲医院。完善计划生育服 务管理，二孩政策全面落实。实施国家、自治区科技项目16项，建成“一园两院三站十三中心”科技创新平台载体，1家企业获自治区主席质量奖、3家企业获市 长质量奖。建成基层文化活动场所237个，《忠勇察哈尔》获自治区艺术创作最高荣誉“萨日纳”奖。自治区“十四运”筹备工作有序推进，全国拳击锦标赛、草 原生态四项、国际马拉松等重大赛事成功举办，我市运动员在全国十三届冬运会上摘得“一金一铜”，实现冰雪项目奖牌“零”的突破。“平安乌兰察布”建设成效 显著。社会矛盾有效化解，刑事和治安案件下降12%和5%。深入开展安全生产大检查百日专项行动，安全生产形势保持稳定。市食药监局被评为全国食药监管系 统先进集体。双拥共建和国防工作深入开展，实现“全国双拥模范城”三连冠。民族团结进步事业开创新局面，宗教领域和谐稳定。人民防空、地震气象、档案史 志、外事侨务等工作得到加强，红十字、妇女儿童、老龄和残疾人事业取得新进展。

——严格依法行政，自身建设不断加强。牢固树立“四个意识”，始终同以习近平同志为核心的党中央保持高度一致。认真贯彻全面从严治党要求，“两学一做” 学习教育深入开展，中央八项规定、自治区和市委配套规定有效落实。制定市政府党组议事决策规则和市级财政资金审批等制度，廉洁建设和反腐败工作全面加强。 公共资金、国有资产、国有资源审计实现全覆盖，公车改革扎实推进，政务公开、行政监察、依法行政工作成效明显。市政府网站连续三年荣获全国政务网站优秀 奖。自觉接受人大法律监督和政协民主监督，认真听取民主党派、工商联和无党派人士意见。提请市人大常委会审议地方性法规议案3件，办理人大代表议案、建议 和政协委员提案476件，办结率100%。

各位代表，过去一年，我们牢记使命、砥砺奋进，战胜了各种风险考验和严峻挑战，取得了令人欣慰的成绩；我们精诚团结、处变不惊，造就了一支对党忠诚、敢 于担当的干部队伍；我们维护大局、协力同心，汇聚起众志成城、无往不胜的磅礴力量。一年的实践启示我们：不管前进的道路多么坎坷，不管面临的困难多么复 杂，只要始终保持正确的政治方向，始终坚持发展第一要务，始终把人民放在心中最高位置，勇于担当、勤奋工作，就一定能够团结带领各族人民创造无愧于心、无 愧于民、无愧于党的新业绩。

各位代表，过去一年的成绩，是自治区党委、政府和市委统揽全局、科学决策的结果，是市人大、市政协有效监督、全力支持的结果，是全市人民同心同德、团结 奋斗的结果。在此，我代表市人民政府，向全市各族人民，向人大代表、政协委员，向各民主党派、工商联、无党派人士和各人民团体，向解放军、武警官兵、政法 干警，向所有关心、支持和参与乌兰察布建设发展的同志们、朋友们，致以崇高的敬意和衷心的感谢!

在总结成绩的同时，我们也清醒地认识到，乌兰察布欠发达的基本市情还没有根本改变，发展中还存在很多困难和问题：经济总量小，结构不合理；项目储备不 足，招商引资力度不够；基础设施薄弱，保障支撑能力不强；城乡居民收入水平不高，脱贫攻坚任务艰巨；生态环境脆弱，一些环境问题还比较突出。政府工作还存 在不足，一些部门依法行政意识不强、行政效能不高，一些干部不重视学习、不研究工作，知识更新不够，主动作为、开拓创新意识不强，思想僵化、动作迟缓，对 新形势新任务研究不够、理解不深，把握和运用政策的能力不强。对于这些问题，我们一定要高度重视，认真加以解决。

二、2017年工作总体安排

2017年，是全面贯彻落实自治区第十次党代会和市第四次党代会精神的第一年，我们将迎来党的十九大胜利召开和自治区成立70周年华诞。做好政府工作， 必须全面贯彻党的十八大和十八届三中、四中、五中、六中全会精神，深入贯彻习近平总书记系列重要讲话和治国理政新理念新思想新战略，认真贯彻习近平总书记 考察内蒙古重要讲话精神，紧紧围绕自治区党委十届二次全会和市委四届二次全会精神，统筹推进“五位一体”总体布局和“四个全面”战略布局，认真践行“五大 发展理念”，坚持稳中求进工作总基调，以推进供给侧结构性改革为主线，突出抓好投资和项目建设，坚决守住发展、生态和民生三条底线，加快推进转型升级，大 力促进“五化协同”，把“围绕首都、依托首都、保障首都、服务首都、得益于首都”作为发展定位，大力实施“三大开放合作战略”，加快发展“四个农牧业优势 产业”，壮大“六个工业支柱产业”，培育“六个新兴服务业”，构筑“六个基地”，打造“三个中心”，坚决打赢脱贫攻坚和五城联创“两个攻坚战”，以优异成 绩庆祝自治区成立70周年，迎接党的十九大胜利召开。

主要预期目标是：地区生产总值增长8%左右；固定资产投资增长13%；社会消费品零售总额增长10%；一般公共预算收入按可比口径增长8.6%；城乡居民人均可支配收入分别增长8%和9%；全面完成国家下达的环境保护约束性指标。

围绕上述目标，我们必须紧紧抓住国家“一带一路”和京津冀协同发展等重大机遇，充分发挥我市区位交通、能源资源、绿色生态等比较优势，用足用好各项政策，解放思想、大胆创新，努力使乌兰察布各项工作“干在实处、走在前列”。重点抓好八个方面的工作。

（一）狠抓投资和项目建设

加大项目推进力度。深入开展“项目建设年”活动，全年计划实施亿元以上项目280个，总投资1914亿元，完成投资540亿元。发挥重大项目绿色审批通 道作用，狠抓项目前期工作，提高审批效率和服务质量，确保按时开工、顺利推进。认真落实项目责任制，建立台账，倒排工期，督查考核，确保项目进度和质量。 紧扣国家政策走向，编制重点项目三年滚动计划，争取更多项目进入国家和自治区重大工程项目包，确保项目前后接续、梯次跟进。

加大招商引资力度。把招商引资作为推动发展的关键一招。强化产业招商。围绕市第四次党代会确定的产业发展定位，有重点、有针对性地招商，着力引进一批带 动行业发展、引领产业升级、补齐发展短板、增强保障能力的大项目、好项目。围绕首都产业转移和功能疏解，加强信息收集研判，重点在大数据、装备制造、木材 加工、服装加工、食品加工、旅游养老、商贸物流等领域取得突破。积极面向环渤海、珠三角、长三角、港澳台开展项目推介。强化政策招商。落实鼓励支持非公经 济发展的政策措施，放宽准入条件，创造非公经济发展的宽松环境。出台金融、物流、旅游等分行业分领域优惠政策，吸引更多企业和项目落户乌兰察布。充分发挥 企业家、商会和乌兰察布籍成功人士的桥梁纽带作用，全方位宣传推介乌兰察布，提高招商成功率。强化环境招商。进一步改善投资环境、创业环境、创新环境，构 建“亲”“清”新型政商关系，营造“亲商、安商、富商”浓厚氛围。认真兑现招商引资政策，提高项目履约率、资金到位率。新官也要理旧账，不以政府换届、领 导人员更替为由违约毁约，树立招商信誉和发展形象。

加大项目融资力度。转变融资观念，打好“财政、银行、证券、保险、担保”组合拳，形成资本服务实体经济、促进产业转型升级的新模式。全面推广PPP模 式。做好项目策划、论证、推介工作，争取更多项目进入国家和自治区PPP项目库。完善风险共担、利益共享等机制，引导社会资本投向市政、交通、生态和公共 服务等领域。加快PPP项目落地实施，形成可复制、可推广的成功经验。推进PPP项目资产证券化，拓宽PPP 项目融资渠道。充分发挥政府引导基金作用。建立与产业导向相衔接的产业发展基金，制定完善相关制度，解决实体企业特别是民营企业融资难问题。推动资源资本 化。做大国源、城投、交投、水投、旅投、华创等公司，整合各行业各领域资源，确立资本化运作模式，增强融资能力。用好支持园区的政策，运用市场化手段，搞 活园区土地开发、融资上市、基础设施建设和招商引资。推动企业上市融资。用足用好国家对贫困地区的IPO扶持政策，制定更加开放优惠的政策措施，支持本地 区企业上市，鼓励外地企业落户我市，利用IPO政策上市。力争年内实现5家企业上市、10家企业进入上市辅导。

（二）着力深化供给侧结构性改革

深入推进“三去一降一补”。坚持分类指导、因城因地施策，有序消化房地产库存。对未建项目用地进行必要调整，对“半拉子”工程按照“一楼一策”，使其尽 快具备交付使用条件，千方百计解决好中心城区拆迁户回迁安置问题。统筹房地产去库存和棚户区改造工作，力争完成棚改任务2.6万套，棚改货币化安置比例达 到90%以上。支持群众改善性住房需求，抑制投机性购房，引导房地产健康发展。坚持标本兼治、综合施策，进一步降低企业负担和融资、物流、用地、用能、制 度性交易成本。引导企业通过强化管理和创新，降低成本、提高效益。坚持既补发展短板也补制度短板，深入实施补短板重点工程，努力在基础设施、脱贫攻坚、产 业转型、民生社会事业等领域取得突破。

深入推进农牧业供给侧结构性改革。扩大优质农畜产品有效供给。围绕老百姓对农畜产品吃得安全、吃得放心的最大关切，制定实施标准化生产技术规程，强化农 畜产品质量安全监管，严把从农田牧场到餐桌的每一道防线。培育乌兰察布特色品牌，新增“三品一标”认证面积30万亩、产品50个，新增地理标志产品2个， 实现农畜产品进京检验前置和结果互认，使直供北京成为乌兰察布农畜产品的金字招牌。打好“互联网+绿色”这张牌，发展电子商务、订单直销等新业态，打造线 上线下相融合的农畜产品营销平台。引进培育一批农牧业领军企业，提高农畜产品精深加工水平，加工转化能力达到60%左右。提升马铃薯产业核心竞争力，编制 完成中国马铃薯之都战略规划，构建繁育、加工、仓储、销售于一体的全产业链，建成国家级马铃薯种薯质量监测检验中心和马铃薯首席专家工作站，供种能力达到 120万吨，加工转化鲜薯160万吨，新增大型智能储窖30万吨。强化冷凉蔬菜基地建设，面积扩大到95万亩，健全冷链物流运输体系，扩大冷凉蔬菜市场占 有率。壮大燕麦杂粮杂豆产业，面积达到165万亩，建成4个燕麦产业基地，开发燕麦系列新产品，申报“中国燕麦之都”。发挥雏鹰、大北农、赛诺等企业的带 动作用，生猪肉鸡肉羊出栏分别达到300万口、1000万羽、1000万只，推动产品就地加工转化增值。深化农村牧区产权制度改革。全面推进土地草牧场确 权登记颁证，推进所有权、承包权、经营权“三权分置”，构建县乡土地草牧场经营权流转平台，鼓励农牧民通过土地草牧场流转、托管、入股等多种形式，发展适 度规模经营。年内流转土地面积100万亩，创建示范合作社100家，培育家庭农牧场示范户50个，农企紧密型利益联结比例达到48%。

（三）推动工业迈向中高端

大力发展新能源产业。抓住国家“光伏领跑者计划”和新能源替代燃煤节能政策，结合矿山环境综合治理和区域微电网建设，推进光伏产业发展，建设光伏产业基 地。新增光伏并网装机50万千瓦。建设吉庆风电基地500万千瓦项目，建成30万千瓦风电供热项目。新增风电并网装机230万千瓦。充分利用陕京四线和西 部天然气管网过境的有利条件，建设一批加工利用项目，日处理能力达到560万立方米。

加快发展大数据产业。抓住内蒙古获批“国家级大数据基础设施统筹发展综合试验区”的契机，完善大数据云计算产业发展规划和政策体系。推进华为数据中心二 期建设，新增服务器10万台。大力发展呼叫外包业务，建成首个蒙语呼叫中心，形成1万个呼叫外包坐席规模。加快建设全市统一的大数据公共服务平台，整合分 散在各级政府各部门各单位的信息平台，建成电子政务协同办公系统。建设医疗云、教育云、环保云和智慧旅游、智慧社区、智慧城管、智慧交通等，形成“大智移 云”生态圈，推动大数据在政用、商用、民用等各个领域应用，着力打造国家智慧城市试点示范城市。

全面提升石墨新材料产业。深化与清华大学合作，借助石墨新材料创新研发中心，加强石墨新产品研发和应用研究，扩大石墨烯等高端新材料生产规模。推进瑞盛石墨等项目建设，建成自治区级石墨检验检测中心和锂离子电池正负极材料生产基地。积极引进发展新能源电池项目。

全力推进高端化工产业。氯碱化工要不断提升PVA、PVC、糊状树脂等中高端产能，推动中联水泥和电石企业合作，发展循环经济。氟化工要加强与科研院所 合作，开发含氟聚合物及其制品、含氟精细化工产品和高性能氟橡胶等系列产品，支持上海三爱富加大研发和深加工力度，着力打造氟化工产业链。

做大做强铁合金产业。推进吉林铁合金等项目建设，延伸产品链条，形成集聚效应，产能增加到700万吨以上，建成全国最大的绿色环保铁合金产业基地。

（四）发展壮大现代服务业

大力发展“物流+”。充分发挥区位交通优势，加快推进现代物流业发展。建设七苏木中欧班列物流基地，加密开行中欧班列，打造面向俄蒙欧的国际物流节点城 市。完善庙梁物流园区功能，发挥博源数字化配煤作用，推进天津港、曹妃甸港在我市建设无水港，继续打造西北煤炭交易中心。构建以天津物产集团为龙头的铁合 金物流，以玫瑰营为基地的药材物流，以康泰惠民园、北京二商等为龙头的农畜产品冷链物流，以新雅宝路市场为龙头的国际商贸物流体系，积极培育第三方物流企 业，为生产企业做好服务配套。建设电子商务跨境产业园和北方食品药品电子商务产业园，提高物流业信息化、智能化、标准化水平。

大力发展“旅游+”。坚持“全域布局、全景打造、全业融合、全民参与”，把全市5.45万平方公里作为一个大景区来规划打造，实现从景点旅游向全域旅 游、四季旅游转变，打响“京郊草原·避暑之都”品牌。提高旅游产品供给质量。引进有实力、有经验的知名企业参与旅游项目投资和经营管理。实施丝路文化博览 园、集宁要塞文化旅游产业园、乌兰哈达火山地质公园、世界动物乐园等26个亿元以上重点项目，打造格根塔拉、辉腾锡勒和岱海温泉等景区升级版。提升旅游城 市形象。加大旅游宣传力度，积极营造“人人都是旅游形象大使、个个都是旅游宣传员、处处都有旅游特色景点”的氛围，各级各部门切实增强为游客服务的意识， 树立乌兰察布旅游新形象。抓好城市及景区公厕、加油加气、供水供电、停车露营、通讯网络、垃圾污水处理等基础设施建设。新建集宁—凉城等10条旅游公路， 提升景区快速通达水平，构建全域旅游交通网。实现中心城区公共场所WIFI全覆盖。建立旅游导视系统和信息服务平台，开发手机APP，发展智慧旅游。推动 旅游业与各产业深度融合。深入挖掘具有地区特色的文化资源，推出精品旅游线路和特色经典剧目，提高游客的参与体验度。加快智慧健康产业孵化园建设，建成百 旺家园医疗养老等项目，打响面向京津的养老养生品牌。开工建设乌兰察布会展中心，积极举办承办中国创业创新博览会、国际木材产业博览会、中蒙博览会分会、 自治区旅游那达慕、首届国际雕塑展等会展活动，提高乌兰察布知名度。以京张冬奥会和自治区“十四运”为契机，积极举办承办“滑向冬奥”环京津冀滑雪邀请 赛、全国羽毛球锦标赛、全国“十三运”拳击选拔赛等16项赛事。加快建设红海子体育产业园等场馆，完成市体育场维修改造和市游泳馆、网球馆、岱海国际滑雪 场一期工程。

大力发展“金融+”。加快市金融综合服务中心建设，吸引银行和证券、保险、基金公司等金融机构入驻我市。加快农信社改制步伐，积极争取设立民营银行、金 融租赁公司、消费金融公司、汽车金融公司等新型金融机构。落实自治区融资担保行业发展三年行动计划，实现各旗县市区国有担保机构全覆盖，打造一个注册资金 10亿元的国有融资担保集团。大力发展普惠金融、绿色金融，实施县域金融工程，引导银行增加信贷投放，努力实现小微企业贷款增速、贷款户数、申贷获得率不 低于上年同期水平，提高金融服务满意度。完善金融风险监测预警和应急处置机制。

（五）加大城乡统筹发展力度

提高城市规划建设管理水平。打赢“五城联创”攻坚战，全面提升城市形象和品位，确保年内成功创建为国家卫生城市、食品安全城市，力争创建为全国文明城 市，积极创建国家环保模范城市、国家生态园林城市，建设环境优美、特色突出、宜居宜业宜游的美丽家园。发挥规划引领作用。突出地域和文化特色，健全市域空 间规划体系，把好山好水好风光融入城市规划建设，避免千篇一律、千城一面。推行“多规合一”，形成统一的规划蓝图和信息管理平台。完善城市功能。坚持地上 地下空间同步开发，新区建设和旧城改造统筹推进，实施水、电、气、暖等市政基础设施建设项目178项。新建各类城市公园绿地13处，新增绿化面积260万 平方米。中心城区绿地率达到37.2%，人均公园绿地面积29.1平方米，主干道两侧建筑全部亮化，白泉山、老虎山、霸王河等景观亮化工程交付使用。实施 老旧住宅小区改造，完成既有建筑节能改造50万平方米以上。加强城市管理。积极探索形式多样的物业管理模式，组建城市管理综合执法局，构建大城管格局，推 进城市管理精细化。

提高城乡一体化发展水平。深化户籍制度改革，加快农村牧区转移人口市民化进程，城镇化率达到50%。大力发展县域经济，打造一批工业重镇、农牧业强镇、 商贸大镇和旅游名镇，创建2个自治区级、10个市级特色小镇。加快美丽乡村建设，巩固农村牧区基础设施和环境建设成果，完成农村牧区危房改造3万户。

提高基础设施网络现代化水平。加快编制“七网”专项规划，形成适应发展、适度超前的基础设施网络体系。建设快速便捷的铁路网。积极推进集大高铁前期工 作，京呼高铁呼和浩特—乌兰察布段开通运营。建设互联互通的公路网。实施重点公路项目33项，建设规模超过3500公里，打通断头路和G6南北通道，呼凉 丰一级公路、国道335集宁至科布尔一级公路在自治区70周年大庆前建成通车。嘎查村通沥青水泥路通畅率达到100%。建设覆盖更广的航空网。推动乌兰察 布机场扩建为国际机场，成为首都国际机场的备降和客货运输协作机场。加密拓展航线航班，年旅客吞吐量达到30万人次。四子王旗、中旗、兴和通用机场完成前 期工作，化德通用机场开工建设，凉城通用机场建成运营。建设高效优质的市政网。新建改建城区主干道146公里、供水管网461公里，集中供热、供水普及率 达到91.5%和85%。启动中心城区河东片区道路管网及综合管廊建设，完成南沙河综合治理。建设安全可靠的水利网。加快丰镇头道沟等3座水库前期工作。 积极推进“引黄济岱”工程。实施中心城区应急水源地工程。建设智能绿色的能源网。加快清洁能源基地及配套电力外送通道前期工作，争取年内获批并开工建设。 积极推进天燃气管网进城入园。建设便捷高效的信息网。加强大数据基础设施建设，建成集宁—北京大容量直通光缆。通过国家电信普遍服务试点项目建设，实现光 纤网络、金融服务、政务服务村村通。建设64个乡级电子商务服务站，856个村级电子商务服务点。建成社会保障等信息比对系统。

（六）全面深化改革开放

推进重点领域改革。深化“放管服”改革，落实“三清单一目录”，把该改的、该减的、该清的、该管的真正落到实处。推进审批事项“两集中、两到位”和网上 并联审批，进一步缩短审批流程和审批时限。完善事中事后监管，实现“双随机一公开”监管全覆盖。深化商事制度改革，加快工商登记全程电子化和简易注销登 记。开展“减证便民”专项行动，全面清理各种证明和手续，最大程度方便群众和企业。深化电力体制改革，扩大电力多边交易和大用户直供规模。深化财税体制改 革，编制财政中期规划，实行预算全过程绩效管理，实现预决算公开。完善财政投资项目评审工作，推进政府采购和购买公共服务。

扩大对外开放合作。加快形成北上南下、东进西出、内外联动、八面来风的对外开放新格局。争取《蒙晋冀（乌大张）长城金三角合作区规划》获得国家批准，组 织召开合作区第四届联席会议。申报建设国家生态安全屏障示范区。积极参与奥运经济，率先在道路联通、文化旅游、冰雪运动、服务保障、农畜产品生产等方面取 得突破，让乌兰察布长久地受益于冬奥会。借助京蒙对口帮扶，与北京市区部门建立紧密型、常态化的对接合作机制，深化与北京在经济、科技、文化、教育、医 疗、扶贫和干部交流等各领域的合作，实现借船出海、借梯登高、借力发展。积极参与和服务中蒙俄经济走廊建设，促进木材、矿产、皮毛等进口资源落地加工。积 极推进善丹呼日勒口岸前期工作，争取乌兰察布机场临时口岸开通运营。

（七）加快建设绿色乌兰察布

加大生态建设和环境保护力度。实施“绿地工程”，完成国家重点生态建设工程43.5万亩，高标准建设G7高速、京呼高铁乌兰察布段、集宁—呼市铁路通道 绿化，完成地方重点区域绿化21.6万亩。严格落实禁牧休牧、草畜平衡制度，加强林木后期管护，巩固生态建设成果。实施“蓝天工程”，加快燃煤锅炉改造， 全面取缔10蒸吨以下燃煤锅炉。完成淘汰黄标车和老旧车任务。加大环境监测监管力度，强化重点行业脱硫脱硝治理。推进中心城区400万平方米煤改电工程， 提高冬季清洁供暖比重。努力实现空气质量优良天数超过310天。实施“碧水工程”，促进岱海、黄旗海水生态修复，启动岱海电厂节水技改，继续实施岱海流域 水改旱工程，加大退水还湖力度。启动霸王河上游综合治理和国家湿地公园建设。

加强工业园区污水处理设施建设，城镇污水集中处理率达到95.3%。实施饮用水水源地保护和饮水安全巩固提升工程，水质达标率和供水保障水平大幅提高。 实施“净土工程”，开展土壤污染详查，全面掌握土壤环境质量状况。划定农用地土壤环境质量类别，强化土壤污染环境执法。

集约节约利用资源。实行最严格的水资源管理和耕地保护制度，强化约束性指标管理，执行能源和水资源消耗、建设用地等总量、强度双控制要求。划定城市发展 边界线、永久基本农田保护红线、生态保护红线，依法用好管好每一寸土地。推动低碳循环发展，促进生产流通消费全过程减量化、再利用、资源化。

加强生态文明制度建设。稳步推进岱海等主要河湖河长制，强化水生态保护。建立项目环评审批与规划环评、区域环境质量联动机制。推行环境污染第三方治理， 加快排污权市场化改革。编制自然资源资产负债表，做好领导干部自然资源资产离任审计试点工作。深化国有林场改革，抓好集体林业综合改革示范工作。积极培育 生态文化和低碳城市建设，推行全民节能行动，倡导绿色低碳健康的生活方式。实施电动汽车充电桩项目。推进城市慢行交通系统建设，在中心城区投放5000辆 公共自行车。

各位代表，清新的空气、宜人的气候、良好的生态是乌兰察布最大的优势。我们要打响空气清新这张牌，把绿色优势转化为发展优势，像保护眼睛一样保护我们的家园，让乌兰察布的天更蓝、山更绿、水更清、空气更清新、人民更开心。（八）全力保障和改善民生

打好脱贫攻坚战。深入实施“五个一批”“六个精准”，年内实现3.7万贫困人口稳定脱贫，凉城县和察右后旗摘掉贫困县帽子。突出产业发展。坚持把发展现 代农牧业作为扶贫脱贫的主渠道，支持与扶贫相关的龙头企业发展壮大，让广大贫困户更多分享农牧业全产业链和价值链增值收益。整合财政涉农涉牧资金，投入 “三到村三到户”扶贫资金2.8亿元，发放金融富民贷款2.4万户、13亿元，充分激发贫困群众脱贫致富的内生动力。推广“菜单式”扶贫和“扶贫+”模 式，将扶贫与电商、旅游、光伏发电、农牧业保险等有机结合起来，根据贫困户能力意愿选择适合发展的特色产业。用好扶贫无息贷款政策，探索资产资本收益扶 贫。推进易地搬迁。对1.7万“一方水土养不起一方人”的贫困人口，通过小村整合和土地流转等方式，分类引导向幸福院、中心村、小城镇和各类园区移民搬 迁、安置就业。年内完成981个村庄的整合任务。实施教育扶贫。做好农村牧区幼儿免费入园工作，实施义务教育学生营养改善计划，1.7万名建档立卡贫困家 庭中小学生实现免费就读。针对产业扶贫对象，开展多形式的技能培训，确保每户有个“明白人”，实现“培训一人、就业一人、脱贫一户”。用好生态扶贫。把贫 困地区符合条件的3500人全部纳入新一轮退耕还林受益范围，6200人优先聘用为护林员，使贫困群众从生态建设和保护中得到实惠。强化兜底保障。将符合 低保条件的扶贫兜底保障对象全部纳入农村牧区最低生活保障范围，其余纳入现金直补范围。对符合缴费条件的建档立卡贫困人口，养老保险每人每年补助100 元。开展精准医疗扶贫，为建档立卡贫困人口进行免费体检，拓宽报销范围，提高门诊报销比例，实现大病商业保险贫困人口全覆盖。创新脱贫机制。开展“万名干 部遍访回访贫困村贫困户”活动，建设“扶贫云”平台，加强脱贫动态管理。完善脱贫退出机制，做好脱贫攻坚考核评估工作。严格落实市县乡村各级各部门脱贫攻 坚责任，深入开展京蒙对口帮扶，积极推进“万企帮万村”行动，形成全社会共同参与的大扶贫格局。

促进创业就业。坚持就业优先战略和更加积极的就业政策，落实高校毕业生就业促进和创业引领计划，支持农村牧区劳动力转移就业和返乡创业，做好军转干部和 退役士兵接收安置工作。年内实现农村牧区劳动力转移就业35万人，城镇新增就业2.5万人。推进大众创业、万众创新，加快建设创客大学、孵化基地、众创空 间等孵化平台，年内市众创大厦投入使用，新增创业平台5个。

完善社会保障体系。实施全民参保计划，健全养老保险参保缴费、转移接续和待遇政策，整合城乡居民医保政策和经办机制，实现就近就地方便快捷办理。建立多 层次保障体系，稳步提高保障对象医疗费用报销比例。统筹城乡社会保障制度，健全社会救助体系，提高重点保障对象补助标准，织密织牢民生保障网。

办好人民满意的教育。继续深化教育“三项改革”，推进教育“管办评”分离改革试点。大力实施标准化学校建设，完成集宁等6个旗县区义务教育均衡发展评估 验收。各旗县市区至少建成2所普惠性幼儿园。提高中小学教育教学质量，打造一批地区名校。加快职业教育改革步伐，开设与大数据运用相关专业，开展学徒制试 点，完成市中等职业技术学校新校区建设和搬迁。优先发展民族教育，支持特殊教育，促进民办教育。推动高等教育转型发展，集宁师范学院通过升本后教育部验 收，职业学院通过自治区示范学校评估。

推进健康乌兰察布建设。全面深化医药卫生体制改革，扎实做好城市公立医院改革工作。促进医疗、医药、医保联动，完善医院法人治理结构，推进分级诊疗制度 建设。完善药品供应保障机制，实现药品零差价，降低药品价格。开展临床路径管理，二级、三级医院临床路径病例数占比达到20%和30%。推行家庭医生签约 服务，签约覆盖率达到30%以上，重点人群达到60%以上。建设市精神卫生医院，启动残疾人康复中心。鼓励规范社会力量办医，落实中蒙医扶持政策。深入开 展爱国卫生运动，做好公共卫生和重大疾病防控工作。深化计划生育服务管理改革，提高出生人口素质。开展全民健身活动，中心城区建成笼式足球场、夜间篮球场 各10个，每个旗县至少建成1个全民健身中心。

公共体育场馆免费或低收费向社会开放。切实保护妇女儿童合法权益，做好关心下一代工作，积极发展残疾人、红十字和老龄事业。

发展科技文化事业。提高企业自主创新能力，加快技术成果转化。实施人才强市战略，积极引进高端人才和创新团队，培养一批实用型技术人才，营造人才实现价 值的良好环境。深化文化体制改革，完善公共文化服务体系、文化产业体系和文化市场体系。推进市大剧院建设，市博物馆、美术馆、科技馆、群艺馆及城市展馆投 入使用。促进艺术创作和演出事业繁荣，举办原创歌曲大赛，创作排演一批有较大影响的剧目，协助完成史诗电视连续剧《英雄出草原》在我市拍摄工作。

建设平安乌兰察布。牢固树立安全生产红线意识，严格落实安全生产责任制，加强信息平台建设，提高安全监管能力。采取政府购买服务的方式，委托专业机构对 企业进行抽查和“会诊”，开展考核评估。全面排除安全隐患，努力减少一般安全事故，坚决防止较大安全事故发生。强化食品安全源头治理和全程监管，迎接国家 食品安全城市中期评估验收，基本建成食品药品安全乌兰察布。巩固提升食品加工园和封闭式农贸市场建设成果，确保各旗县食品小作坊入园率和流动摊贩入市率达 到80%以上。推动社区、社会组织、社会工作者“三社联动”，提高城乡社区治理服务水平，实现城镇社区场所全部达标，20%的嘎查村建成农村社区。创新信 访工作机制，有效调处化解矛盾纠纷。推进公安科技信息化建设，新建高清视频监控2000个，建成全市应急联动平台，构建立体化社会治安防控体系，严密防范 和依法惩治各类违法犯罪，增强人民群众的安全感。支持国防建设和军队改革，做好双拥和人防工作，推动军民深度融合发展。

深入开展民族团结进步教育和创建活动，全面贯彻党的宗教工作基本方针。做好统计、质监、地震、气象、档案等工作，推动社会全面进步。

各位代表！为人民服务，对人民负责，受人民监督，让人民满意，是政府工作坚守的信念。面对新任务、新要求，我们将更加重视自身建设，把全面建成小康社会 使命扛在肩上，把万家忧乐放在心头，建设人民满意的政府。一要忠诚担当。进一步增强“四个意识”特别是核心意识、看齐意识，坚定自觉地同以习近平同志为核 心的党中央保持高度一致，把对党忠诚、对国家忠诚、对人民忠诚、对事业忠诚体现到实际工作中，认真履职尽责，敢于担当、主动作为，以更严的要求、更高的标 准推动工作。贯彻落实正向激励和容错纠错机制，真正使想干事的有机会、会干事的有舞台、能干事的有地位。二要依法行政。坚持法无授权不可为、法定职责必须 为，把政府工作纳入法治轨道。加强政府法制工作，严格规范性文件备案审查，完善法律顾问和公职律师制度。全面落实行政执法责任制，推进行政执法能力建设， 促进规范公正文明执法。落实“七五”普法，强化法律援助，维护群众合法权益。自觉接受人大法律监督、政协民主监督、社会舆论监督，认真办理人大代表议案、 建议和政协委员提案，广泛听取各民主党派、工商联、无党派人士意见，积极支持工会、共青团、妇联等群众团体开展工作。三要服务大局。牢固树立“大服务”的 理念，为广大群众、投资客商以及全社会搞好服务，以服务推动发展、引导投资、促进建设。深入实施“互联网+政务服务”等信息惠民工程，丰富网上服务内容， 完善网上办事流程，让信息多跑路，让群众和企业少跑腿、好办事。推进政务公开，增强政府工作透明度。建立新闻发布会制度，及时回应社会关切。加强诚信政府 建设，提高政府公信力和执行力。四要廉洁从政。深入开展党风廉洁建设和反腐败斗争，坚决惩治和查处损害群众利益的不正之风和腐败问题。坚持勤俭节约，严控 “三公”经费。加大审计监督和廉政风险防控力度，强化行政监察，严肃查处各类违纪违法案件，坚决整治庸政懒政怠政行为。五要真抓实干。大力发扬“马上就 办”的精神、钉钉子的精神和“蒙古马”精神，提高政府执行力和行政效率，想事、谋事、干事、成事，不务虚功、不图虚名。对新政策、新知识、新形势，认真学 习、准确掌握、自觉运用，始终站在时代前沿思考问题、推动工作。对重点项目、重大事项、重要工作，勤谋划、快上手、抓落实，用干部的辛苦指数换取人民的幸 福指数。

各位代表！发展全凭实干，奋斗成就梦想。让我们更加紧密地团结在以习近平同志为核心的党中央周围，在自治区党委、政府和市委的坚强领导下，守望相助、团 结奋斗，苦干实干积极干、撸起袖子加油干，奋力谱写乌兰察布跨越发展新篇章，以优异的成绩向自治区成立70周年献礼，以崭新的面貌迎接党的十九大胜利召开！
